# Supplementary material for: Laboratory earthquakes decipher control and stability of rupture speeds
Source: Nat Commun. 2023 Apr 27;14:2427. doi: 10.1038/s41467-023-38137-w (PMC10140064; doi:10.1038/s41467-023-38137-w)
Supplement: Supplementary file 1 — Supplementary Information [file 41467_2023_38137_MOESM1_ESM.pdf]

Laboratory earthquakes decipher control and stability of rupture speeds

Authors: Peng Dong[1], Kaiwen Xia[1,2,3]*, Ying Xu[3], Derek Elsworth[4], Jean-Paul Ampuero[5]

[1]Institute of Geosafety, China University of Geosciences (Beijing), Beijing 100083. China

[2]Department of Civil and Mineral Engineering, University of Toronto; Ontario, Canada M5S 1A4.

[3]State Key Laboratory of Hydraulic Engineering Simulation and Safety, School of Civil Engineering, Tianjin University, Tianjin 300072, China.

[4]Energy and Mineral Engineering & Geosciences, G3 Center and EMS Energy Institute, Pennsylvania State University; University Park, Pennsylvania 16802, USA.

[5]Géoazur, Université Côte d'Azur, IRD, CNRS, Observatoire de la Côte d'Azur; 250 rue Albert Einstein, 14 Sophia Antipolis, 06560 Valbonne, France

*Corresponding author. Email: Kaiwen Xia (kaiwen.xia@utoronto.ca)

This document presents:

-Supplementary Figures 1 to 9

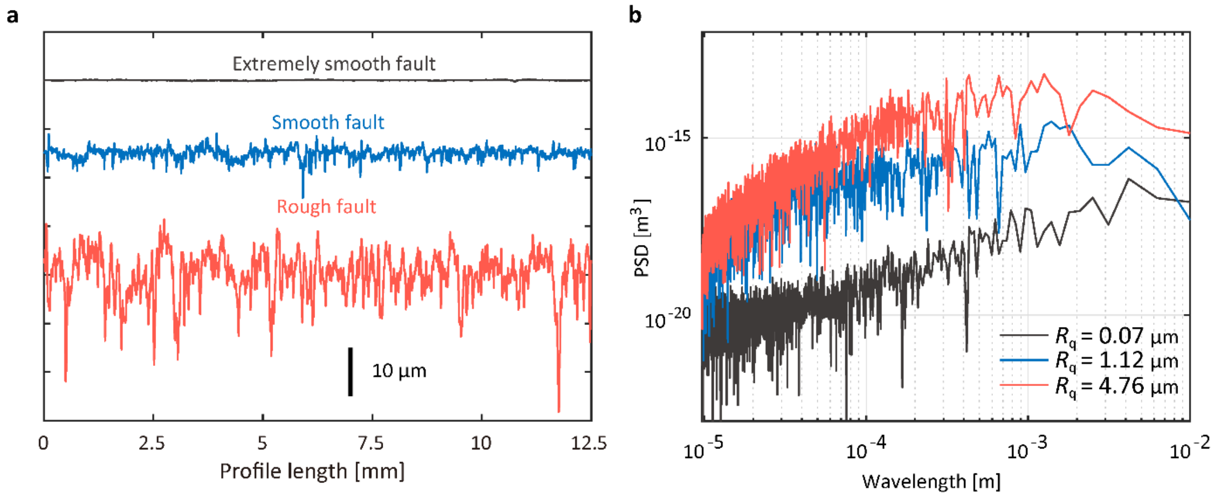

Supplementary Figure 1. Typical surface topography of laboratory faults. a, Profiles and **b**, power spectral density for the extremely smooth fault, smooth fault, and rough fault.

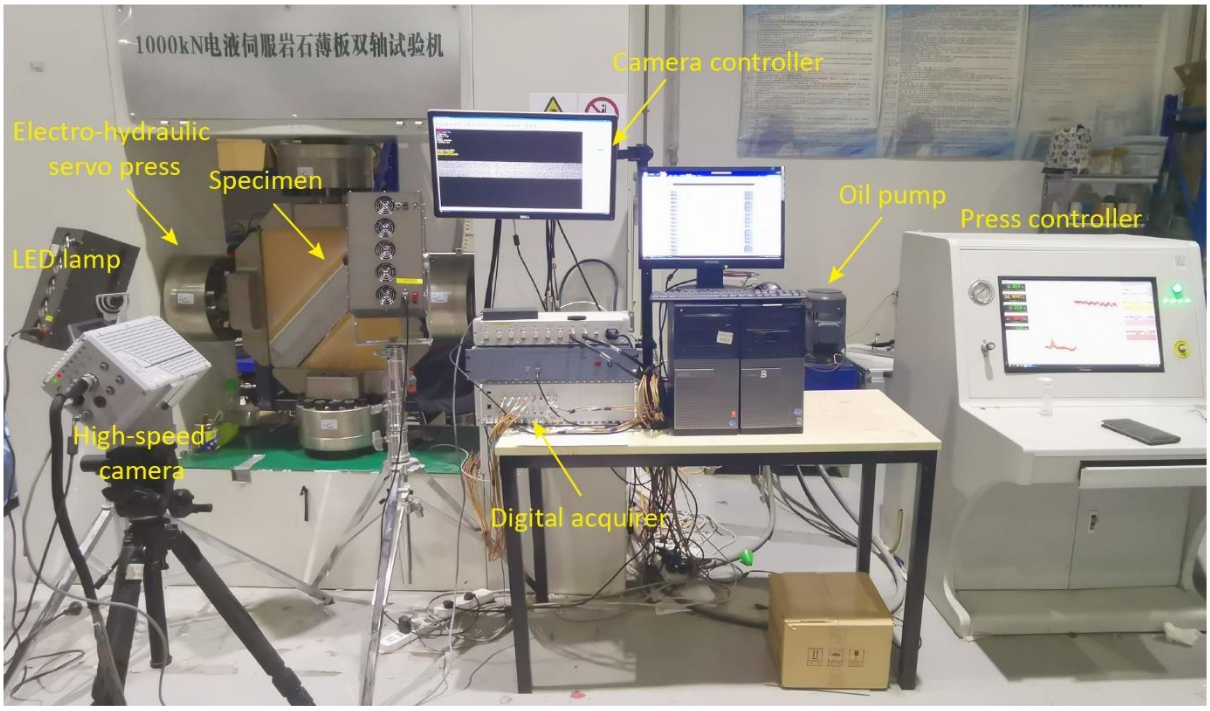

Supplementary Figure 2. Laboratory earthquake setup. The otherwise square PMMA plate contains a straight interface that mimics geological fault. The specimen is biaxially loaded by an electro-hydraulic servo frame. With increase of the differential stress, the fault may spontaneously rupture. A Photron FASTCAM SA1.1 camera is used to capture textured images of the specimen region enclosing the fault during the rupture nucleation and propagation. Multi-point strain gauge array is used to measure the shear strain at seven selected locations along the fault.

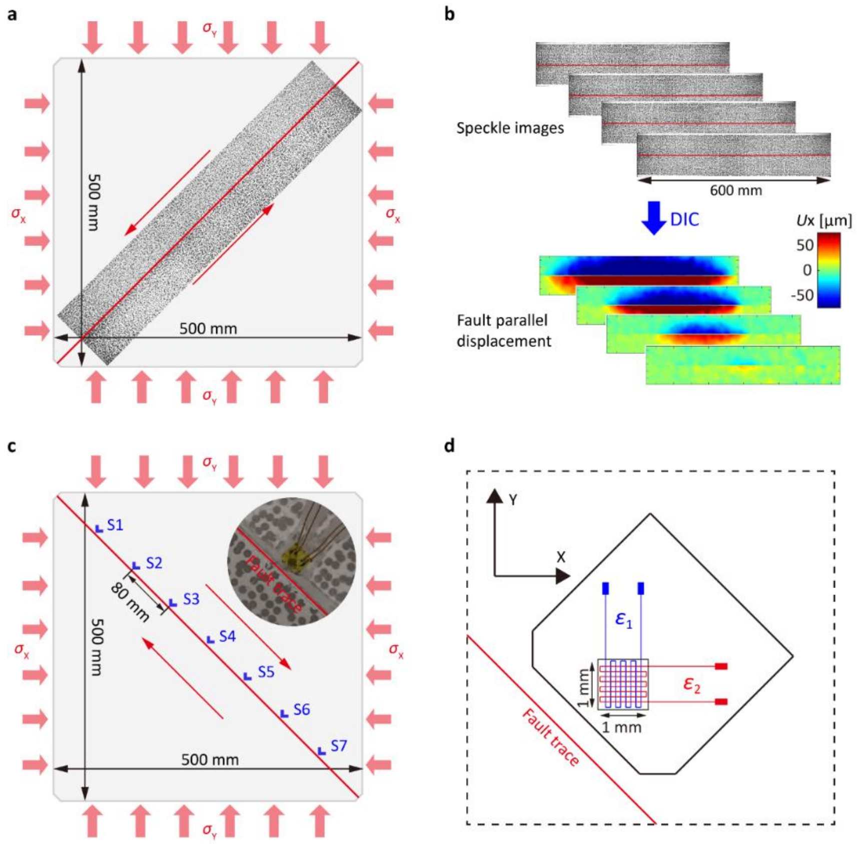

Supplementary Figure 3. Diagnostic methods. a, Frontside of the fault model. The fault trace is represented as a red line. The specimen surface is coated with speckle patterns. **b**, Evolution of fault parallel displacement field is obtained by the DIC method. **c**, Backside of the fault model. A series of shear strain gauges are mounted along the fault line to monitor the shear strain near the fault. Insert shows the photograph of the mounted shear strain gauge (the background speckle is on the other side of the transparent specimen). **d**, The schematic drawing of the shear strain gauge, whose effective length is 1 mm.

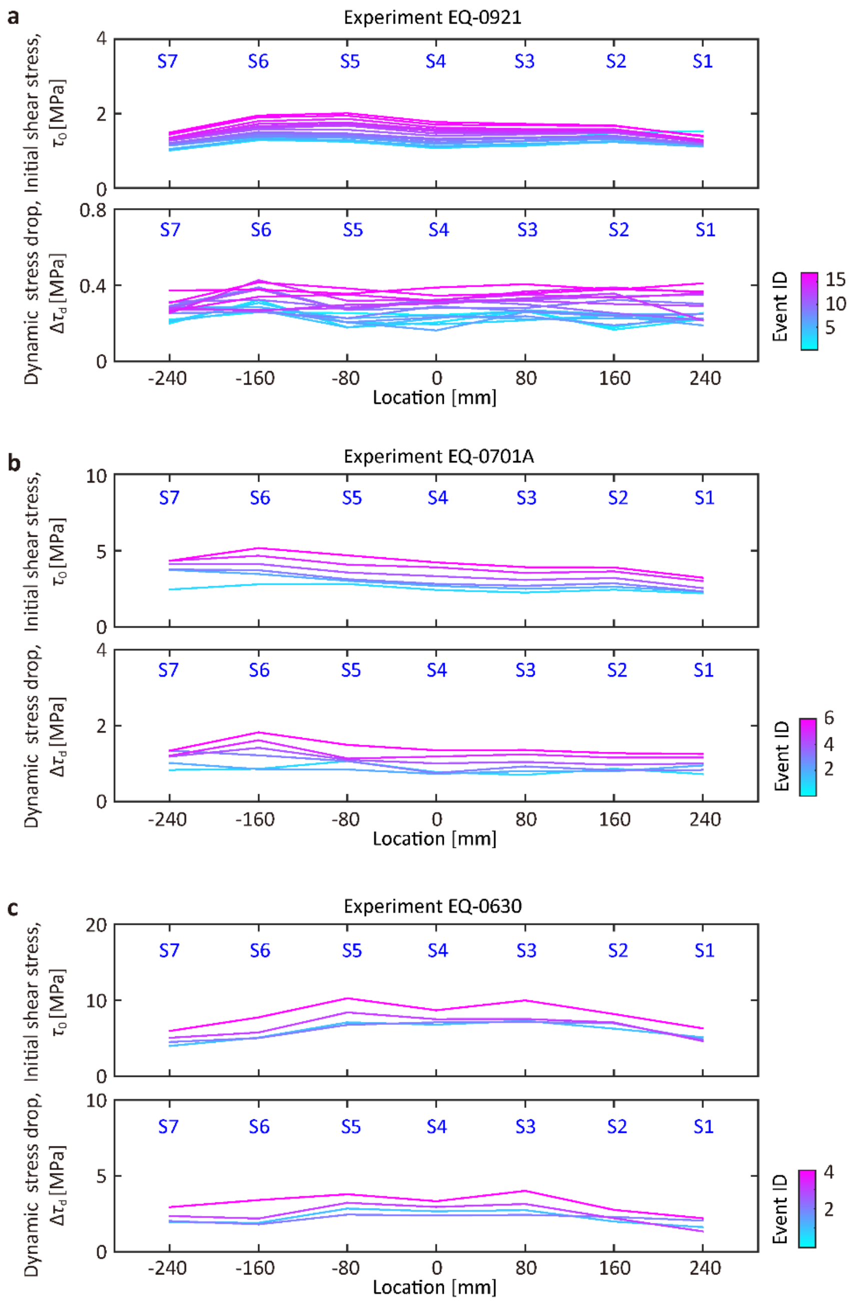

Supplementary Figure 4. Distribution of initial shear stress τ_0 and dynamic stress drop $\Delta\tau_d$ for three sequences of stick-slip events. a, Stick-slip events on extremely smooth fault with σ_X = 2 MPa. **b,** Stick-slip events on smooth fault with σ_X = 2 MPa. **c**, Stick-slip events on rough fault with σ_X = 3 MPa**.** Initial shear stress and dynamic stress drop are measured by strain gauges (S1~S7) mounted along the fault. Initial shear stress is the value measured at about 1 ms before the onset of the instability. When the sample is not subjected to loading, the shear stress is considered as zero for reference. The color of each line is coded with event ID in the stick-slip sequence.

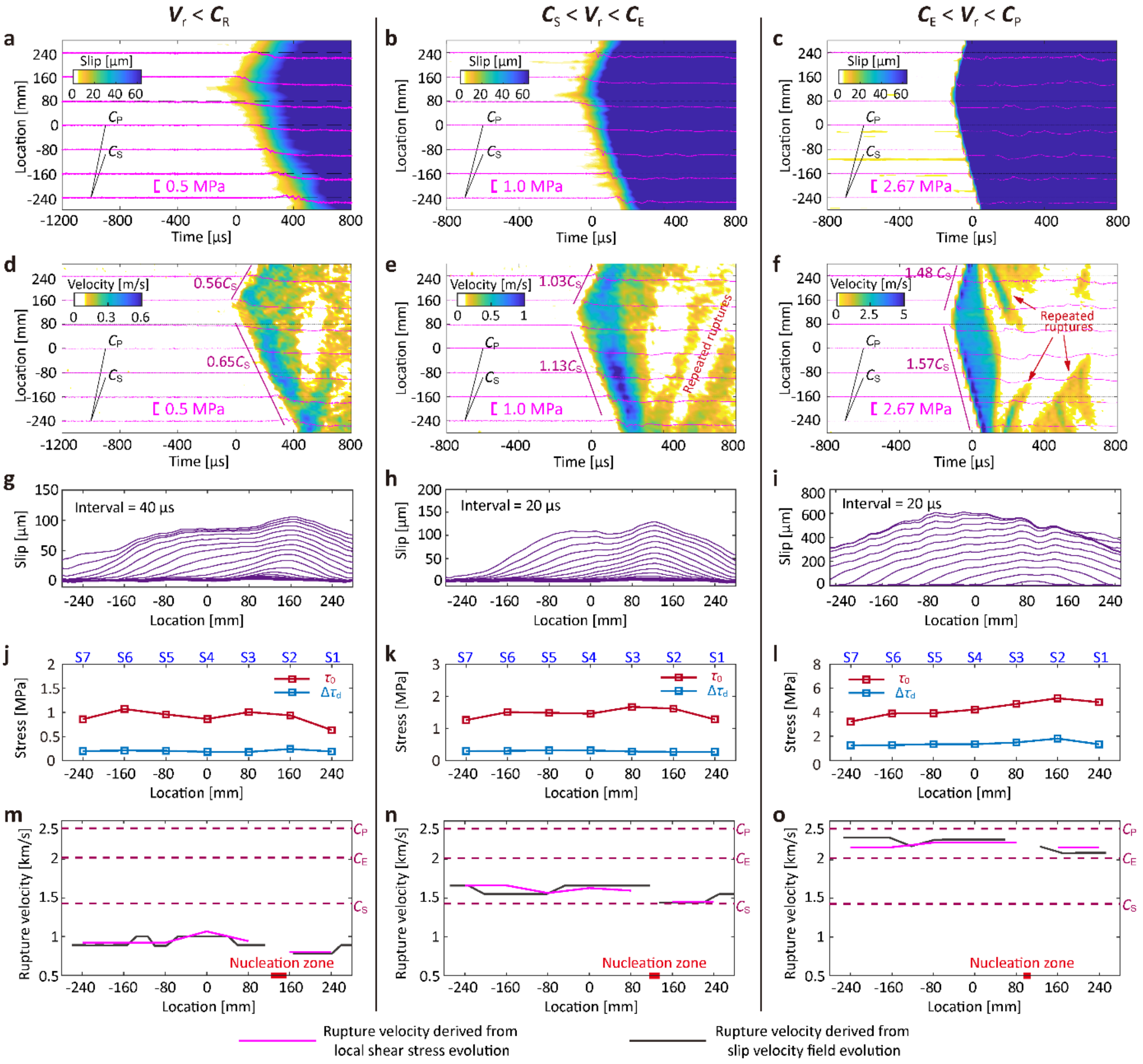

Supplementary Figure 5. Three events with different rupture speeds elucidate the rupture dynamics of laboratory earthquakes. a, **b**, **c**, Spatio-temporal evolution of fault slip and evolution of local shear stress for (**a**) subRayleigh (**b**) subEshelby and (**c**) supershear ruptures. **d**, **e**, **f**, Spatio-temporal evolution of fault-parallel slip velocity and evolution of local shear stress for the same events as in **a**, **b**, and **c**. **g**, **h**, **i**, Slip profile validates the crack-like sustained rupture for the same events as in **a**, **b**, and **c**. **j**, **k**, **l**, Distribution of initial shear stress τ_0 and dynamic stress drop for the same events as in **a**, **b**, and **c**. **m**, **n**, **o**, Rupture velocity along the faut for the same events as in **a**, **b**, and **c**. The nucleation zone is defined by the red rectangle.

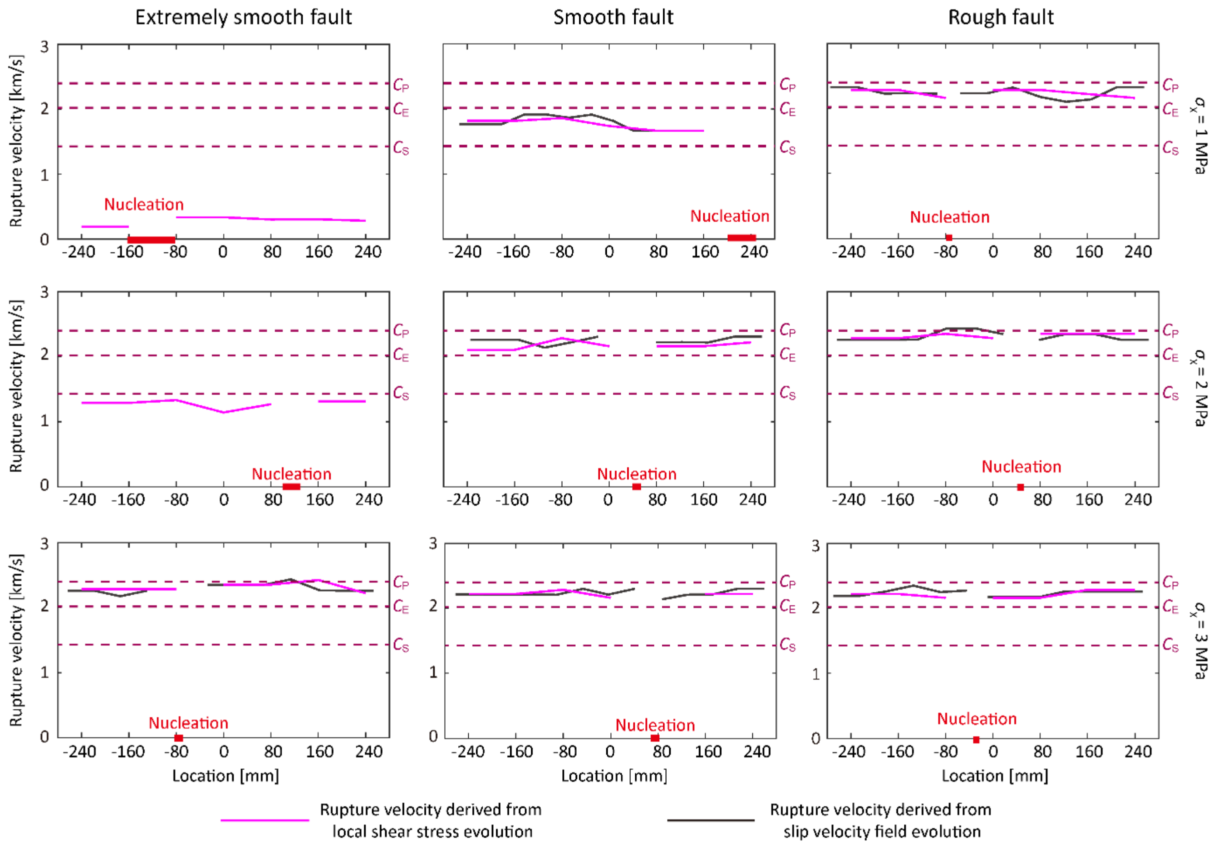

Supplementary Figure 6. Evolution of rupture velocity along the fault for events shown in Fig. 2. The rupture velocity derived from the full-field measurement of fault slip and local shear strain evolution are shown as magenta and black lines. The nucleation zone is defined by the red rectangle. For the events with low resolution in slip velocity, only the rupture speed derived from strain signals is presented.

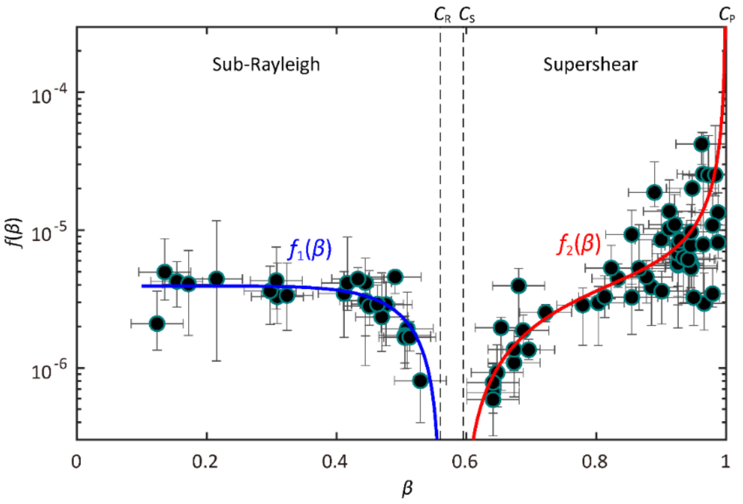

Supplementary Figure 7. Determination of the function $f(\beta)$ by fitting laboratory data. Blue curve represents the fitted function $f_1(\beta)$ for the subRayleigh events and red curve represents the fitted function $f_2(\beta)$ for the supershear events. The horizontal error bars indicate the variation in rupture velocity along the strike for each event. The vertical error bars indicate the range of $f(\beta)$ calculated from dynamic stress drop with small variations along the strike.

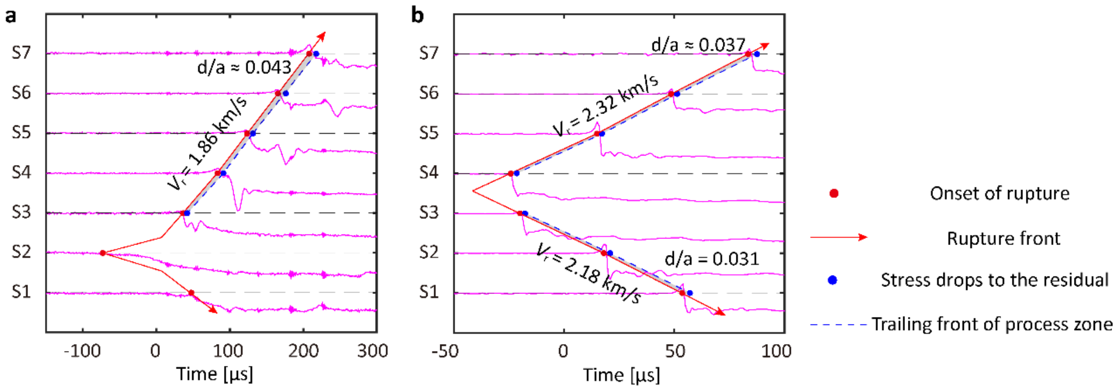

Supplementary Figure 8. Estimation of the ratio between the process zone size *d* and the rupture length *a*. a, **b** show the evolution of shear strain (magenta curves) of two typical cases of supershear ruptures. The rupture process zone is indicated as shaded area.

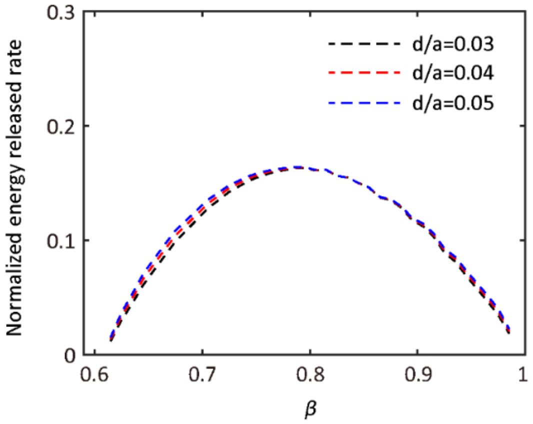

Supplementary Figure 9. Normalized energy release rate as a function of *β* following Broberg's analysis. The results of *d*/*a* = 0.03 (black), 0.04 (red), and 0.05 (blue) are presented. The minor change in *d*/*a* does not produce a significant change in the normalized energy released rate.
