# Supplementary material for: Laboratory earthquakes decipher control and stability of rupture speeds
Source: Nat Commun. 2023 Apr 27;14:2427. doi: 10.1038/s41467-023-38137-w (PMC10140064; doi:10.1038/s41467-023-38137-w)
Supplement: Supplementary file 3 — Description of Additional Supplementary Files [file 41467_2023_38137_MOESM3_ESM.pdf]

Description of Additional Supplementary Files

Supplementary Data 1
Description: Testing conditions and results for all experiments.

Supplementary Data 2
Description: Source parameters of the strike-slip earthquakes compiled from SRCMOD.

Supplementary Data 3
Description: Rupture velocities and stress drops on the fast-rupture segments of natural faults.
